# Advances in metabolic engineering of *Corynebacterium glutamicum* to produce high-value active ingredients for food, feed, human health, and well-being

**DOI:** 10.1042/EBC20200134

**Published:** 2021-07-26

**Authors:** Sabrina Wolf, Judith Becker, Yota Tsuge, Hideo Kawaguchi, Akihiko Kondo, Jan Marienhagen, Michael Bott, Volker F. Wendisch, Christoph Wittmann

**Affiliations:** 1 Institute of Systems Biotechnology, Saarland University, Saarbrücken, Germany; 2 Institute for Frontier Science Initiative, Kanazawa University, Japan; 3 Graduate School of Science, Technology and Innovation, Kobe University, Japan; 4 Graduate School of Engineering, Kobe University, Japan; 5 Biomass Engineering Research Division, RIKEN, Yokohama, Japan; 6 Institute of Bio- and Geosciences, IBG-1: Biotechnology, Forschungszentrum Jülich, Germany; 7 Institute of Biotechnology, RWTH Aachen University, Aachen, Germany; 8 The Bioeconomy Science Center (BioSC), Forschungszentrum Jülich, Germany; 9 Genetics of Prokaryotes, Biology & CeBiTec, Bielefeld University, Germany

**Keywords:** Corynebacterium glutamicum, extremolytes, metabolic engineering, plant phenols, synthetic biology, terpenoids

## Abstract

The soil microbe *Corynebacterium glutamicum* is a leading workhorse in industrial biotechnology and has become famous for its power to synthetise amino acids and a range of bulk chemicals at high titre and yield. The product portfolio of the microbe is continuously expanding. Moreover, metabolically engineered strains of *C. glutamicum* produce more than 30 high value active ingredients, including signature molecules of raspberry, savoury, and orange flavours, sun blockers, anti-ageing sugars, and polymers for regenerative medicine. Herein, we highlight recent advances in engineering of the microbe into novel cell factories that overproduce these precious molecules from pioneering proofs-of-concept up to industrial productivity.

## Introduction

*Corynebacterium glutamicum* is a Gram-positive, non-spore-forming facultative anaerobic bacterium with a moderate to high GC content belonging to the phylum of actinobacteria [1]. The microbe is traditionally used to manufacture amino acids through fermentation [2], including the premium products l-glutamate [3,4], l-lysine [5–7], l-arginine [8], and l-tryptophan [9]. Remarkable efforts in metabolic engineering have widened the product portfolio of *C. glutamicum* to over 70 different compounds [10], including bulk biofuels [11,12], and bulk chemicals such as lactate [13,14], succinate [13,15,16], *cis,cis*-muconate [17], cadaverine (diaminopentane) [18–21], aminovalerate [22], glutarate [22–25], and 3-amino-4-hydroxybenzoate [26]. The lessons learned about *C. glutamicum* have revealed: (i) the microbe grows quickly to high cell densities, shows no autolysis, and can be easily propagated to a large scale (≥750 cubic meters). (ii) *C. glutamicum* produces no endotoxins, does not undergo phage lysis, and is generally recognised as safe (GRAS), allowing the synthesis of a range of commercial products granted GRAS status by the United States Food and Drug Administration for the food and pharmaceutical industries. (iii) It consumes various carbon substrates and can simultaneously utilise substrate mixtures [27–29], favouring the application of hydrolysed lignocellulosic biomass and even waste materials as eco-friendly feedstock for fermentation [11,17,27,30]. (iv) *C. glutamicum* shows a high tolerance to toxic compounds and other forms of stress [31] due to its robust cell wall, composed of a thick glycan core and, in some strains, a crystalline surface S-layer [32–34].

Supported by a well-established understanding of its genomic repertoire [31,33], powerful engineering tools and techniques have been developed to systematically analyse and modify *C. glutamicum*. Systems-wide (multi)omics approaches [35–37] have allowed experimental studies of the microbe at transcriptome, proteome, metabolome, and fluxome levels [10,38] and their functional interactions [37,39], while computational simulations have explored the metabolic pathway capabilities and optimum flux states, and guiding strain engineering [40–42]. For tailored gene expression, a wide range of low- and high-copy number shuttle vectors, promoters, and control elements, selection markers, and reporter genes are available [33,43–48]. These techniques have, *inter alia*, enabled the construction of genome reduced strains such as *C. glutamicum* C1*, CR099, and their derivatives, which have emerged as a valuable chassis for basic research and industrial development [49,50]. Other interesting developments have involved the use of biosensors to translate intracellular product levels into optical outputs to screen for superior phenotypes [51–53]. Evolutionary approaches have provided strains with elevated tolerance to industrial processing environments, including high temperatures [54] and oxidative stress [55], which are important from a production standpoint. Recent efforts have also enabled the use of *C. glutamicum* in anodic electro-fermentations [56].

**Figure 1:**
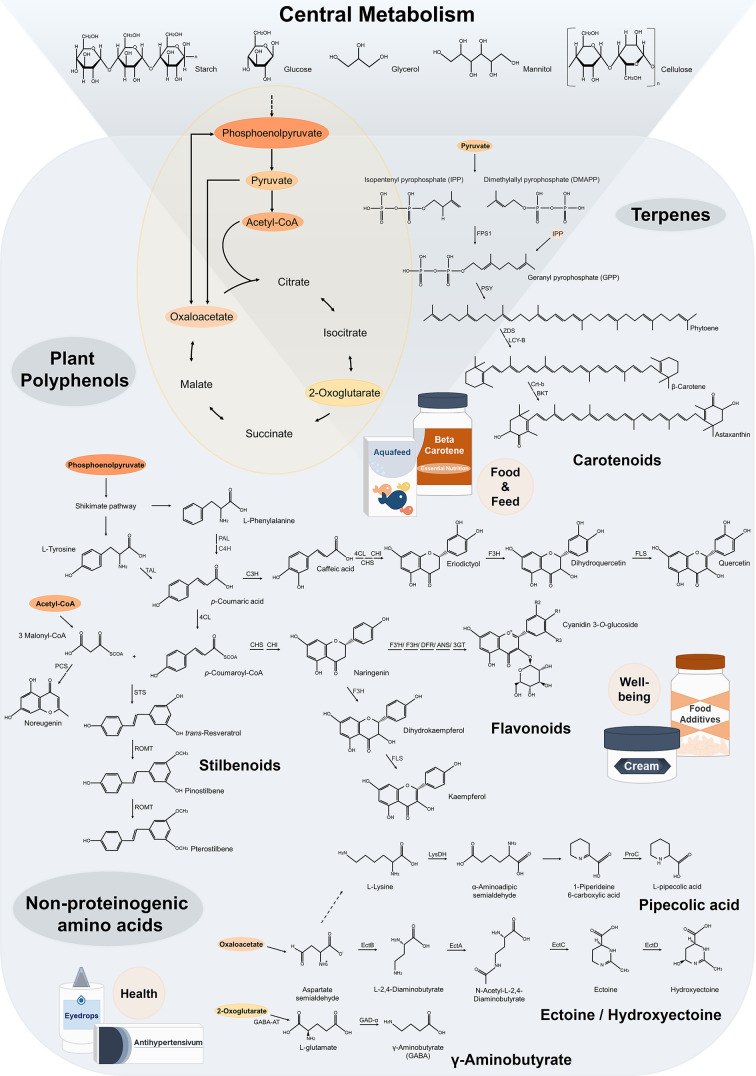
Metabolic pathway map illustrating high-value bioactive ingredients for food, agricultural feed materials, human health, and well-being products, provided by *Corynebacterium glutamicum* cell factories

Herein, we review recent achievements in the metabolic engineering of *C. glutamicum* for the production of high-value molecules to be used in medical, pharmaceutical, and nutraceutical applications, including amino acids, (poly)phenols, terpenoids, extremolytes, and medical polymers (Figure 1), extending from valuable proof-of-concept studies up industrial processes (Tables 1-4).

**Table 1:** Fermentative production of high-value branched chain and non-proteinogenic amino acids using metabolically engineered *C. glutamicum*

Product	Genotype	Substrate	Titre [g.l^−1^]	Productivity [g.l^−1^ h^−1^]	Yield [g g^−1^]	Reference
l-Valine	**R (JCM 18229)**^a^					
	Δ*ldhA* Δ*ppc* Δ*pta*					
	Δ*ackA* Δ*ctfA*					
	Δ*avtA_ilv*N^GE^C™ +	Glucose	150.0	6.3	0.57	[63]
	*gapA* + *pyk* + *pfkA* +					
	*pgi* + *tpi* + pCRB-					
	BN^GE^C™ + pCRB-DLD					
l-Leucine	**ML1-9**^b^					
	*ΔilvA ΔalaT Δldh*	Glucose	38.1	0.8	0.30	[69]
	*ΔltbR ΔpanBC*					
	*leuA^R^*					
l-Isoleucine	**IWJ001**^c^					
	+ pDXW‐8‐*gnd*‐*fbp*‐*pgl*	Glucose	29.0	0.4	0.14	[70]
γ-Aminobutyrate	**G01**					
	*gad plk, ΔargB ΔproB ΔdapA*	Glucose	70.6	1.0	n.s.^d^	[78]

*^a^ilvN*^GE^, feedback-resistant mutant *ilvN* (G156E); *ilvC*™, NAD-preferring mutant *ilvC* (S34G, L48E, R49F).

b ML1-9, classical mutant from screening against *l-*leucine analogues, *leuA^R^*, feedback resistant mutant *leuA* (R529H, G532D, L535V).

c IWJ001, industrial *l-*isoleucine producer.

d n.s., not shown.

**Table 2:** Fermentative production of high value (poly)phenols, and related natural products using metabolically engineered *C. glutamicum*

Product	Genotype	Substrate/Precursor	Titre [mg.l^−1^]	Reference
Cyanidin 3-O-glucoside (C3G)	+ pZM1-eftuSUMO-3GT-eftuANS	Catechin	40	[88]
Salidroside	**MB001(DE3)**			
	DelAro^4^ C5 *mufasO_BCD1_*	Tyrosol	9000	[86]
	+ pMKEx2_*malE-OsUGT13*			
Naringenin	**MB001(DE3)**			
	DelAro^4^-4cl_Pc_	*p*-Coumaric acid	37	[96]
	+ pMKEx2 _chs*_Ph_* _chi*_Ph_*			
	+ pEKEx3_f3h*_Ph_* _fls*_Pd_*			
Dihydrokaempferol	**MB001(DE3)**			
	DelAro^4^-4cl_Pc_	*p*-Coumaric acid	20	[96]
	+ pMKEx2 _chs*_Ph_* _chi*_Ph_*			
	+ pEKEx3_f3h*_Ph_* _fls*_Pd_*			
Kaempferol	**MB001(DE3)**			
	DelAro^4^-4cl_Pc_	*p*-Coumaric acid	23	[96]
	+ pMKEx2 _chs*_Ph_* _chi*_Ph_*			
	+ pEKEx3_f3h*_Ph_* _fls*_Pd_*			
Eriodictyol	**MB001(DE3)**			
	DelAro^4^-4cl_Pc_	Caffeic acid	12	[96]
	+ pMKEx2 _chs*_Ph_* _chi*_Ph_*			
	+ pEKEx3_f3h*_Ph_* _fls*_Pd_*			
Dihydroquercetin	**MB001(DE3)**			
	DelAro^4^-4cl_Pc_	Caffeic acid	7	[96]
	+ pMKEx2 _chs*_Ph_* _chi*_Ph_*			
	+ pEKEx3_f3h*_Ph_* _fls*_Pd_*			
Quercetin	**MB001(DE3)**			
	DelAro^4^-4cl_Pc_	Caffeic acid	10	[96]
	+ pMKEx2 _chs*_Ph_* _chi*_Ph_*			
	+ pEKEx3_f3h*_Ph_* _fls*_Pd_*			
Resveratrol	**MB001(DE3)**			
	DelAro^3^	*p*-Coumaric acid	158	[96,97]
	+ pMKEx2_			
	*sts_Ah_*_4cl*_Pc_*			
Mono-O-methylated pinostilbene	**MB001(DE3)**			
	DelAro^4^			
	+ pMKEx2_	*p*-Coumaric acid	3	[96]
	*sts_Ah_*_ 4cl*_Pc_*			
	+ pEKEx3_			
	malE*_Ec_*-omt*_Vv_*			
Di-O-methylated pterostilbene	**MB001(DE3)**			
	DelAro^4^			
	+ pMKEx2_	*p*-Coumaric acid	42	[96]
	*sts_Ah_*_ 4cl*_Pc_*			
	+ pEKEx3_			
	malE*_Ec_*-omt*_Vv_*			
Pinosylvin	**MB001(DE3)**			
	DelAro3 +pMK2_*sts_Ah_*_*4cl_Pc_*	Cinnamic acid	121	[97]
Piceatannol	**MB001(DE3)**			
	DelAro3 +pMK2_*sts_Ah_*_*4cl_Pc_*	Caffeic acid	56	[97]
Noreugenin	**MB001(DE3)**			
	DelAro^4^-4cl*_Pc_*	Glucose	53	[94]
	C5 mu*fas*O*_BCD1_* P*_O6_-iolT1 ∆pyc*			
	*+* pMKEx2-*pcs_AaCg_*-short			
Raspberry ketone	**MB001(DE3)**			
	DelAro^4^-*4cl_Pc_* C5 mu*fas*O*_BCD1_*	*p*-Coumaric acid	100	[100]
	P*_O6_-iolT1 ∆pyc ΔldhA*			
	+ pMKEx2-*bas_RpCg_-curA_EcCg_*			
	+ pEKEx3-*udhA_EcCg_*			
Zingerone	**MB001(DE3)**			
	DelAro^4^-*4cl_Pc_* C5 mu*fas*O*_BCD1_*			
	P*_O6_-iolT1 ∆pyc ΔldhA*	Ferulic acid	70	[100]
	+ pMKEx2-*bas_RpCg_-curA_EcCg_*			
	+ pEKEx3-*udhA_EcCg_*			
Benzylacetone	**MB001(DE3)**			
	DelAro^4^-*4cl_Pc_* C5 mu*fas*O*_BCD1_*			
	P*_O6_-iolT1 ∆pyc ΔldhA*	Cinnamic acid	11	[100]
	+ pMKEx2-*bas_RpCg_-curA_EcCg_*			
	+ pEKEx3-*udhA_EcCg_*			
6-Methlysalicylate	**MB001(DE3)**			
	DelAro^4^-*4cl_Pc_* C5 mu*fas*O*_BCD1_*	Glucose	41	[103]
	+ pMKEx2_*malE_Ec_-chlB1_Sa_*			

MB001 is a variant prophage-free, genome reduced strain of *C. glutamicum* [146], while MB001(DE3) is a derivative of MB001 with the addition of a chromosomally encoded T7 based expression system [147]

**Table 3:** Fermentative production of terpenoids using metabolically engineered *C. glutamicum*

Product	Genotype	Substrate	Titre [mg.l^−1^]	Productivity [mg.l^−1^ h^−1^]	Reference
*Astaxanthin*	ASTA*	Glucose	22	0.46	[107]
*(+)-Valencene*	**VLC6**	Glucose	41	1.7	[112]
*Patchoulol*	**PAT3**	Glucose	60	0.42	[110]
*CoQ10*	**UBI413**	Glucose	0.4	0.004	[113]

**Table 4:** Fermentative production of high-value extremolytes using metabolically engineered *C. glutamicum*

Product	Genotype	Substrate	Titre [g.l^−1^]	Productivity [g.l^−1^ h^−1^]	Yield [g g^−1^]	Reference
*Ectoine*	**ATCC 13032** *lysC^fbr^ ectABC^opt.^*	Sugar and molasses	65.2	1.2	0.19	[119]
*Hydroxyectoine*	**ECT-2**	Glucose	0.4	-	-	[123]
*α-Glucosylglycerol*	**Δ*otsA* IMglgA** + pEKEx3-*ggpSP*	Sucrose	2.1	-	0.14	[129]
*l-Pipecolic acid*	**PIPE 4**	Glucose and sucrose	14.4	0.2	0.20	[128]

## Pharmaceutical amino acids

Amino acids are essential for health and play an important role as food additives, medically active ingredients, and building blocks for pharmaceuticals [57]. In 2020, the global amino acid market reached a volume of 9.8 million tons per year, which is estimated at US$ 21 Billion and is projected to reach US$ 27 Billion in 2027. The top low-price bulk amino acids for use in food and feed are l-lysine [6], l-glutamate [3], l-tryptophan [9], and l-methionine [41,58]. In addition, *C. glutamicum* has been successfully engineered to produce amino acids with a higher value, mainly for pharmaceutical and medical applications, including branched chain and non-proteinogenic derivatives [59] (Table 1).

### The branched chain amino acids l-valine, l-leucine, and l-isoleucine

The amino acid l-valine is an important precursor of antibiotics [60,61] and herbicides [62]. Engineered *C. glutamicum* strains accumulate l-valine to a titre of 227 g.L^−1^ under oxygen deprivation within only 48 h [63]. Although the reported value was corrected for volume increase and dilution effects during fed-batch production and the effective concentrations reached during the process were approximately 2-fold lower [64], the achieved performance is undoubtedly impressive (Table 1). Producing strains are characterised by enhanced biosynthesis controlled by acetohydroxyacid synthase (AHAS, *ilvN^GE^*), disrupted routes to undesired by-products (lactate, succinate), an amplified glycolytic pathway, and optimised redox balancing using an NAD-preferring mutant of acetohydroxyacid isomeroreductase (AHAIR, *ilvC*™) during production [57,60]. Transcriptomics and proteomics furthermore have revealed that up-regulation of the branched chain amino acid exporter genes *brnFE* promotes l-valine secretion capability [65], displaying a promising target for future metabolic engineering interventions [66].

Furthermore, it was also possible to generate l-leucine-producing strains of *C. glutamicum* characterised by the overexpression of feedback-resistant variants of the central enzyme 2-isopropylmalate synthase (IPMS, *leuA*) and a fine-tuned redistribution of precursor supply [57,67]. For example, overexpression of a mutated *leuA* variant (amino acid exchanges R529H, G532D) was combined with deletion of *ltbR* (*leuBCD*), PTS-independent glucose uptake via *IolR* deletion, and an attenuated flux through citrate synthase (*gltA*), strategies yielding a strain that achieved 24 g.l^−1^ l-leucine within 72 h in a fed-batch process [68]. The best performance was achieved by metabolic engineering of a classically generated mutant strain (ML1-9), obtained by screening of structural l-leucine analogues [69]. The producer overexpressed a feedback resistant *leuA* gene variant with three amino acid exchanges (R529H, G532D, and L535V) but lacked *ltbR* to increase expression of *leuBCD* and lacked *alaT, panBC*, and *ilvA* to increase precursor availability (Table 1). It achieved a titre of 38.1 g.L^−1^ and l-leucine production, which using this strain, could achieve quantities up to industrial scales (150 m^3^) [69].

Interesting engineering strategies transformed deregulated l-lysine producers into l-isoleucine producers. As an example, the expression of several gene copies of *hom*, encoding a feedback-resistant l-homoserine dehydrogenase, and *ilvA*, encoding a deregulated l-threonine dehydratase, yielded 13 g.l^−1^ l-isoleucine [68]. Metabolic engineering of the industrial l-isoleucine producing strain IWJ001 channelled carbon into the pentose phosphate pathway for enhanced NADPH supply and increased the production up to 29 g.l^−1^ l-isoleucine [70].

### The neurotransmitter γ-aminobutyrate

γ-Aminobutyrate (GABA) is a non-proteinogenic amino acid of four carbons, which exhibits recognised blood pressure lowering activity and is used in pharmaceuticals and functional foods [71–73]. Biochemically, it is derived from l-glutamate via pyridoxal phosphate (PLP) dependent l-glutamate decarboxylase (GAD), suggesting *C. glutamicum* with its known l-glutamate production potential as promising host, although the microbe is also able to use GABA as sole carbon and nitrogen source [74]. Heterologous expression of l-glutamate decarboxylase (*gadAB*) from *Escherichia coli* in wild-type *C. glutamicum* ATCC 13032 yielded a producer which accumulated 8 g.l^−1^ GABA within 96 h [72]. Subsequently, the impact of GABA catabolism and re-uptake was assessed, leading to the discovery of the GABA specific transport protein GabP [75]. A *gabP* deletion mutant showed 12.5% higher GABA production than the parental strain. Expression of a mutant l-glutamate decarboxylase with a broader pH optimum under the control of a strong synthetic promoter (H36) then leveraged production to 38.1 g.l^−1^ [76]. Subsequently, additional expression of *xylA* from *E. coli* provided strains that simultaneously utilised glucose and xylose and accumulated GABA up to a titre of 35.5 g.l^−1^, using an empty fruit bunch biosugar solution as a carbon source [77]. An elegant approach decoupled GABA production from the requirement for external PLP, an otherwise expensive ingredient [78]. The cofactor was simply regenerated by expressing PLP kinase from *Lactobacillus plantarum* GB 01-21. Combined with heterologous expression of GAD from the same donor and disruption of pathways to relevant by-products (l-arginine, l-proline, l-lysine), this strategy has yielded the best GABA producer to date (Table 1). The engineered *C. glutamicum* strain achieved 70.6 g.l^−1^ GABA [78]. An alternative pathway to GABA was established in putrescine-producing *C. glutamicum* by expressing the *E. coli* genes putrescine transaminase *(patA*) and γ-aminobutyraldehyde dehydrogenase *(patD*), which enabled a titre of 5.3 g.l^−1^ [79].

### The microbial sunscreen shinorine

Shinorine (mycosporine-glycine-serine) belongs to the group of mycosporine and mycosporine-like amino acids which are small, water soluble compounds with a cyclohexenone or cyclohexenimine scaffold. Naturally, these molecules are synthesised by cyanobacteria, fungi, and in micro- and macroalgae. They efficiently absorb UVA and UVB light, capture reactive oxygen species (ROS), protect macromolecules and cells and act as microbial sunscreens, which are promising for skin care cosmetics [80,81]. The biosynthesis of shinorine requires the intermediate sedoheptulose 7-phosphate which is converted to dimethyl 4-deoxygadusol and 4-deoxygadusol, and is followed by the addition of a glycine to form mycosporine-glycine and the final attachment of l-serine, involving a non-ribosomal peptide synthetase (NRPS) homologue [81]. Heterologous shinorine production in *C. glutamicum* therefore involves a modification of the pentose phosphate pathway to increase sedoheptulose 7-phosphate supply, specifically, the deletion of transaldolase (*tal*) and the overexpression of 6-phosphogluconate dehydrogenase (*gnd*) [81]. Plasmid-based expression of the shinorine operon genes (*amir4256-amir4259*) from *Actinosynnema mirum* then yielded the compound at a titre of 19 mg.l^−1^ [81].

## Plant polyphenols

Plant polyphenols are naturally found in fruits, vegetables, cereals, and beverages [82–84], and comprise several thousand compounds [83,85]. Studies suggest that long-term consumption of a diet rich in plant polyphenols offers protection against different cancers, cardiovascular diseases, diabetes, osteoporosis, and neurodegenerative diseases [82–84], opening opportunities for the application of polyphenols as pharmaceuticals, nutraceuticals, and food additives [86]. Microbial production normally outcompetes traditional extraction from plant tissues, given drawbacks such as low yield, seasonal variation, and product instability, associated to the latter [87,88]. In comparison with other tested hosts, *C. glutamicum* shows superior natural robustness against toxic aromatics [89], making it a promising host for the production of aromatic molecules [88,90,91] (Table 2). This trait can be partially attributed to the complex catabolic network for aromatic compounds in *C. glutamicum*, which needs to be modified prior to establishing polyphenol biosynthesis in this organism [91,92]. Furthermore, because plant polyphenol synthesis requires large amounts of malonyl-CoA [93], the central metabolism of *C. glutamicum* was extensively re-engineered to meet the increased demands for this metabolite [94,95]. Additional challenges arose from the fact that implementation of pathways for polyphenols synthesis requires the functional expression of plant-derived enzymes in bacteria, which are often characterised by incomplete posttranslational modification or formation of inclusion bodies due to incorrect protein folding [82–84,86,88,90,96,97].

### Flavonoids

Flavonoids are characterised by two benzene rings linked to a pyrane or pyrone ring, and derivatives result from substitution with hydroxyl groups, including alkylated and/or glycosylated moieties [88,96]. Recently, several flavonoids were successfully produced in recombinant *C. glutamicum* [88,96,97] (Table 2). For example, the production of the anthocyanin cyanidin 3-O-glucoside from catechin was achieved by co-expression of anthocyanidin synthase from *Petunia hybrida* and 3-O-glucosyltransferase from *Arabidopsis thaliana* enabling a production titre of 40 mg.l^−1^, when UDP-glucose was supplied [88].

### Stilbenoids

Stilbenoids are hydroxylated diarylethenes, characterised by an ethylene moiety with one phenyl group on each side. Most stilbenoids are synthesised in plants as response to infection or injury. Studies suggest that resveratrol, the most prominent and commercially relevant stilbenoid, mediates health-promoting effects against a range of different cancers and supports the immune system and antioxidative defence mechanisms [90]. The first resveratrol producing *C. glutamicum* variants carried genes for 4-coumaroyl-CoA ligase (4CL) obtained from *Petroselinum crispum* and stilbene synthase (STS) from *Arachis hypogaea* and allowed an accumulation of up to 158 mg.l^−1^ of resveratrol when supplemented with *p*-coumaric acid [96,97] (Table 2). Alternatively, a synthetic reverse β-oxidative pathway was established in *C. glutamicum*, which allowed the synthesis of resveratrol from inexpensive 4-hydroxybenzoate without supplementation of the much more costly *p*-coumaric acid [98]. Furthermore, resveratrol can be directly produced from glucose without addition of any precursor molecules. However, in addition to the heterologous expression of genes conferring 4CL and STS activity, these strains required a tyrosine ammonia-lyase activity for the non-oxidative elimination of the primary amino group of l-tyrosine provided by microbial metabolism [97,99].

## Plant phenols

### Salidroside, active against neurodegenerative diseases

A success story in bacterial plant phenol synthesis is the production of salidroside, a compound found in many raspberry species and active against the pathological processes of neurodegenerative diseases [84,86]. In a recombinant *C. glutamicum* variant, a titre of 9 g.l^−1^ salidroside from supplemented tyrosol was achieved by improving UDP-glucose supply and the heterologous expression the glycosyltransferase gene from *Oryza sativa* [84,86] (Table 2). Furthermore, three flavouring phenylbutanoids raspberry ketone, zingerone, and benzylacetone, can be synthesised by supplementing phenylpropanoid precursors using an engineered *C. glutamicum* strain [100]. The key to success was the functional implementation of the curcumin reductase CurA from *Escherichia coli* which possesses unknown benzalacetone reductase activity, required for phenylbutanoid synthesis.

### 6-Methylsalicylate, a flavouring agent

*Corynebacterium glutamicum* was also engineered for the synthesis of 6-methylsalicylate (6-MSA), the methyl ester of salicylic acid found in many plant species, particularly wintergreens [101]. Despite applications as a fragrance or flavouring agent, 6-MSA can also be used in high concentrations as an analgesic and rubefacient to treat joint and muscular pain [102]. The key to the synthesis of 41 mg.l^−1^ 6-MSA from glucose with *C. glutamicum* is the functional expression of 6-MSA synthase ChlB1 from *Streptomyces antibioticus* [103], which is a larger (186 kDa) type I polyketide synthase (Table 2).

## Terpenoids

Terpenoids comprise carotenoids that are natural yellow- to red-coloured pigments found in plants, fungi, algae, and bacteria [11,71]. They function as light-harvesting photo protectants, membrane stabilisers, and hormone precursors [11]. Chemically, terpenoids consist of isoprene units and are classified according to the length of their carbon backbone [71]. Due to their beneficial effects on humans and in animal health, in particular due to their antioxidative properties, terpenoids are applied as pharmaceuticals and nutraceuticals in the healthcare industry [11,104].

### Carotenoids

Most carotenoids contain 40 carbon atoms [105]. The two most prominent representatives are β-carotene, a precursor of pro-vitamin A [105] and astaxanthin, one of the most abundant marine carotenoids and one of the strongest natural antioxidants [106,107]. Naturally, *C. glutamicum* contains the glycosylated C50 carotenoid decaprenoxanthin as a yellow pigment and, correspondingly, carotenoid biosynthetic genes of the non-mevalonate pathway, involving isopentenyl pyrophosphate as a central intermediate [104]. Native carotenoid biosynthesis is controlled by the GGPP-responsive transcriptional repressor CrtR [108] and is increased via CrtR upon exposure to light [109]. Recent efforts have enabled the production of carotenoids in *C. glutamicum* with, including the use of lycopene cyclase (*crtY*) obtained from *Pantoea ananatis* allowing the production of β-carotene (Table 3). Upon additional heterologous expression of β-carotene ketolase (*crtW*) from *Brevundimonas aurantiaca* and hydroxylase (*crtZ*) (*P. ananatis*) the recombinant strain produced astaxanthin at a rate of 0.4 mg.l^−1^ h^−1^, providing a promising alternative to current algae-based production [106]. Translational fusion of the membrane proteins CrtW with CrtZ improved the production of astaxanthin by 7-fold [107].

### Sesquiterpenes

The C15 sesquiterpenes are volatile, which contributes to their use as flavouring agents and fragrances. (+)-Valencene and patchoulol are fragrances present in plant essential oils. Replacement of the endogenous GGPP synthase by *E. coli* FPP synthase combined with heterologous expression of the plant patchoulol synthase gene from *Pogostemon cablin* enabled the production of 60 mg.l^−1^ patchoulol [110] (Table 3). For (+)-valencene production, expression of codon-optimised valencene synthase from the cedar *Callitropsis nootkatensis* [111] was used instead of patchoulol synthase and upon the use of photocaged IPTG as an optogenetic switch the growth-inhibiting (+)-valencene could be produced to 41 mg.l^−1^ [112].

### Coenzyme Q10

Coenzyme Q10 (CoQ10) serves as an electron carrier in aerobic respiration and exerts antioxidative effects when used as supplements in patients with various diseases. It is an interesting compound. *C. glutamicum* was the first microbe not natively synthesising CoQ10 that was engineered for CoQ10 production [113] (Table 3). A carotenoid-deficient strain with increased supply of the precursor FPP was constructed to synthesise decaprenyl diphosphate (DPP) and was the first CoQ10 precursor to express the DPP synthase gene *ddsA* isolated from *Paracoccus denitrificans.* Metabolic engineering of the shikimate pathway provided para-hydroxybenzoate (pHBA) as the second CoQ10 precursor. Using *ubi* genes from *E. coli* allowed the prenylation of pHBA with DPP followed by decarboxylation, hydroxylation, and methylation reactions to yield CoQ10 [113].

## Pyrazines

Pyrazines are monocyclic aromatic rings with two nitrogen atoms, widely used as flavouring agents. *C. glutamicum* is capable of synthetising these molecules endogenously [114]. Feeding experiments with deuterated acetoin resulted in the incorporation of ^2^H labelling in tri-methylpyrazine and tetra-methylpyrazine [114]. Together with specifically created *C. glutamicum* deletion mutants these experiments allowed elucidation of the biosynthetic pathways that produce pyrazines in detail. More recently, heterologous strains of *C. glutamicum*, which expressed mevalonate kinase from *S. aureus* and *C. kroppenstedtii* and 3-hydroxy-3-methylglutaryl-CoA reductase from *S. aureus*, allowed the synthesis of up to 5 g.l^−1^ tetra-methylpyrazine [115].

## Extremolytes

Extremolytes are small molecules, found in extremophilic bacteria and archaea [68]. They are crucial for adapting their lifestyle to hot, sour, or salty environments due to their protective properties [116,117]. They stabilise and protect macromolecules, membranes, cells, and tissues [118]. Typically, extremolytes are active against different stresses, making them multi-functional agents for the cosmetic, medical, and food industries [116,117]. Chemically, extremolytes comprise a diverse group and include sugars, polyols, heterosides, amino acids, and their derivatives.

### The industrial flagship extremolyte small molecule ectoine

Ectoine (S-2-methyl-1,4,5,6-tetrahydropyrimidine-4-carboxylic acid) is the industrial flagship molecule among extremolytes [119,120]. Discovered in the halophilic bacterium *Halorhodospira halochloris* [116,117], ectoine has been produced at a scale of several tons per year and achieves prices up to of 1000 US$ kg^−1^ [121]. Its applications include the preservation and protection of the skin against cell damage and aging, treatment of atopic dermatitis, lung inflammation, allergic rhinitis, and Alzheimer’s disease, and acts to stabilise proteins, DNA, and RNA [119,120]. Presently, the halophilic microbe *Halomonas elongata* is used for the industrial production of ectoine. The established ‘bacterial milking’ process is based on intracellular ectoine accumulation in high-salt medium (15% salt), followed by transfer of the cells to a low salinity solution (3% salt), which then promotes ectoine release due to an osmotic down-shock reaction [116]. In addition to the costly industrial process, corrosive damage to conventional stainless steel fermenters and connected devices caused by the salt concentrations have recently stimulated the development of low-salt production strategies [119,120]. Thereby, *C. glutamicum* has emerged as the world’s best microbial ectoine producer (Table 4). Pioneering studies have engineered the overproduction of ectoine (plus its derivative hydroxyectoine) in a strain of *C. glutamicum*, which expressed the *ectABCD* operon from *Pseudomonas stutzeri* [119], and relied on a biosynthetic involving pathway aspartate semialdehyde as the central precursor. The use of a constitutive promoter decoupled gene expression from its native control by high osmolarity [121,122] and the deletion of the lysine exporter abolished l-lysine as a by-product [119]. The tailored strain ECT-2 achieved an ectoine tire of 4.5 g.l^−1^ from glucose without the use of a high-salinity medium [123]. Subsequently, metabolic engineering of alternative pathways supplied elevated levels of aspartate-semialdehyde, de-repressed glucose metabolism, and abolished lactate secretion [124], which were all beneficial for ectoine formation. The obtained mutant *C. glutamicum* Ecto-5 achieved an ectoine titre of 22 g.l^−1^ together with 6 g.l^−1^ l-lysine as a by-product [124]. More recently, ectoine production could be enhanced even further. For this purpose, the conventional polycistronic operon design was replaced by a monocistronic design to individually control the expression of each of the three genes [119]. A library of 185,193 possible variants of synthetic pathways under randomly distributed expression control was transformed into a chassis strain *C. glutamicum*, which expressed a feedback resistant aspartokinase to increase supply of aspartate-semialdehyde but lacked the l-lysine exporter to prevent l-lysine secretion. Screening of hundreds of clones finally yielded the strain *C. glutamicum ect^opt^*, which formed 65 g.l^−1^ ectoine in a fed-batch process, almost without any by‐products [119].

### L-Pipecolic acid, a pharmaceutical building block and cell protectant

l-Pipecolic acid (piperidine 2-carboxylic acid, PA) serves as chiral building block for therapeutic agents [125], and recently its value as a cell-protecting compatible solute has been revealed [126]. Because pipecolic acid is derived from l-lysine, l-lysine overproducers were engineered for l-pipecolic acid production by a synthetic pathway involving oxidative deamination, dehydration, and reduction by l-lysine 6-dehydrogenase (deaminating) from *Silicibacter pomeroyi* and endogenous pyrroline 5-carboxylate reductase [127] (Table 4). Upon abolishment of the export of the precursor l-lysine and the improvement of expression of the pathway genes, l-pipecolic acid was produced to a titre of 14.4 g.l^−1^ [128]. The cell-protective properties of l-pipecolic acid as an osmo-protectant could also be demonstrated for the recombinant *C. glutamicum* strain [126].

### Glucosyl-glycerol, an anti-aging sugar derivative

Glucosyl-glycerol (glycoin, R-2-O-α-d-glucopyranosyl-glycerol) is naturally produced in marine cyanobacteria and has promising anti-aging activity for use in cosmetics and pharmaceuticals. A recent study described glucosyl-glycerol overproduction in recombinant *C. glutamicum* [129]. For this purpose, a two-step biosynthetic pathway using the cyanobacterium *Synechocystis* spp. was introduced. Interestingly, production occurred only in osmotically stressed cells. The elimination of routes to trehalose and glycogen synthesis, competition for ADP-glucose, and nitrogen-limiting conditions finally allowed a α-glucosyl-glycerol titre of 2 g.l^−1^ to be achieved (Table 4). This process displayed a promising proof-of-concept but work is needed to achieve the high performance of a highly selective enzyme catalytic process, which is now used industrially for α-glucosyl-glycerol manufacturing [116].

## Hyaluronic acid

Hyaluronic acid is a naturally occurring polymer. It consists of linear chains of double units of d-glucuronic acid (GlcA) and *N*-acetyl-d-glucosamine (GlcNAc) with more than 30,000 repeats [130,131] and plays an important role in maintaining structural integrity of cells, tissues, and body fluids [132]. Hyaluronic acid exerts various medical properties. It has been used as a surgical aid in ophthalmology, for joint disease, and wound healing, including skin and cartilage repair [131,133,134]. The market value is estimated at US$10 billion [133]. To overcome the inherent limitations of classical hyaluronic acid extraction from rooster combs and bovine eyes with regards to product safety, reproducibility, and costs [130], significant efforts have been made to develop microbial-based production. The first attempts involved the use of the natural producer *Streptococcus bacterium*, and provided 7 g.l^−1^ hyaluronic acid [130] but presented the disadvantage of the potential pathogenicity of the strain [133]. More recently, low levels of hyaluronic acid production have been established in *E. coli* (3.8 g.l^−1^), *Lactococcus lactis* (1.8 g.l^−1^), and *Bacillus subtilis* (6.8 g.l^−1^) [133,134]. Recently, *C. glutamicum* revealed remarkable hyaluronic acid production capacity. By engineering and evaluation of eight different organisations of the hyaluronic acid operon *hasABCDE* and further strain optimisation, it was possible to produce 28.7 g.l^−1^ of hyaluronic acid from glucose in a fed-batch fermentation process [133–135].

## Conclusions and outlook

*C. glutamicum* has emerged as a potent host to produce molecules for human health and well-being, and has greatly expanded its role from a traditional producer of amino acids, chemicals, and materials to a multi-functional microbial production platform. Different success stories have been highlighted in this study, which demonstrate the product capacity of chemically diverse and complex molecules for high-value applications, using entire pathways or synthetic pathway assemblies from various organisms (Tables 1-4). In addition to amino acids, plant (poly)phenols, terpenoids, extremolytes, and medical polymers, have been showcased here. *C. glutamicum* was recently shown to produce antibiotics such as roseoflavin [136], vitamins such as d-pantothenate [137] and vitamin B_2_ [138], and diagnostic biomarkers for the characterisation of various cancer types such as l-2-hydroxyglutarate [139], *N*-alkylated amino acids such as *N*-methyl-l-alanine [140], and *N*-ethyl-sarcosine [141], as well as chlorinated or brominated l-tryptophans [142,143] for the synthesis of peptide drugs, promising an even wider portfolio in the future. In addition, the functional expression of type I polyketide synthase, renders *C. glutamicum* a promising microbial cell factory to produce type I polyketide synthase-derived high-value molecules. Its valuable native and engineered traits, low nutritional requirements, and capacity for producing chemicals at high titre and yields from second [39] and third generation renewables [144,145], and simultaneous use of sugar mixtures [29], together with a demonstrated robustness [17] ensure the establishment of simple manufacturing processes using *C. glutamicum* all over the world. In the near future, we can expect a further widening of the product portfolio as well as the establishment of next-level cell factories with increased titres, yields, and rates towards an accelerated commercialisation.

## Summary

- *Corynebacterium glutamicum* is an efficient cell factory for high-value natural products.
- More than 30 accessible compounds have been developed.
- Applications include food, feed, cosmetics, and medical industries.
- Production is enabled by efficient expression of synthetic pathways.

## Abbreviations

4CL: 4-coumaroyl-CoA ligase
DPP: decaprenyl diphosphate
NRPS: non-ribosomal peptide synthetase
pHBA: para-hydroxybenzoate
ROS: reactive oxygen species
STS: stilbene synthase
